# Analysis of a Fractional-Order Couple Model with Acceleration in Feelings

**DOI:** 10.1155/2013/730736

**Published:** 2013-12-12

**Authors:** Ilknur Koca, Nuri Ozalp

**Affiliations:** ^1^Department of Mathematics, Faculty of Sciences, Gaziantep University, 27100 Gaziantep, Turkey; ^2^Department of Mathematics, Faculty of Sciences, Ankara University, 06100 Ankara, Turkey

## Abstract

A fractional-order nonlinear dynamical model of couple has been introduced. Upper bounds are obtained for a fractional-order nonlinear dynamical model. Also different from other models, a model with the order 2**α** is discussed. We are expecting an acceleration in feelings; that is why we increase the order of the derivative between 1 < 2*α* ≤ 2. Stability analysis of the fractional-order nonlinear dynamical model of involving two persons is studied using the fractional Routh-Hurwitz criteria. By using stability analysis on fractional-order system, we obtain sufficient condition on the parameters for the locally asymptotic stability of equilibrium points. Finally, numerical simulations are presented to verify the obtained results.

## 1. Introduction

The first noninteger order differentiation and integration notion was considered in 1695 by Leibniz and L'Hôpital. In a letter to L'Hôpital in 1695, Leibniz raised the following question: “Can the meaning of derivatives with integer order be generalized to derivatives with noninteger orders?” L'Hôpital was somewhat curious about that question and replied by another question to Leibniz: “What if the order will be 1/2?” After the letter was answered by Leibniz, fractional order in the concept of derivative was formed [1].

There are lots of topics on fractional modeling, but in recent decades the study of interpersonal relationships has begun to be popular. Interpersonal relationships appear in many contexts, such as in family, kinship, acquaintance, work, and clubs [2]. Mathematical modeling in interpersonal relationships is very important for capturing the dynamics of people, but there are few models in this area and models have been limited to integer order differential equations. Another interesting dynamic is marriage. Marriage has been studied scientifically for the past sixty years [3]. Researchers are trying to understand why some couples divorce, but others do not, and why, among those who remain married, some are happy and some are miserable with one another [4]. Since experiments in these areas are difficult to generate, mathematical models may play a role in explanation of the dynamics of a couple and behavioral features.

Recently, a fractional-order system for the dynamics of love affair between a couple has been considered [5]. In this paper, different from [5], a model with the order 2*α* is discussed. We are expecting an acceleration in feelings; that is why we increase the order of the derivative between 1 < 2*α* ≤ 2. Also, upper bounds are discussed for the system.

We begin by giving the definitions and properties of fractional-order integrals and derivatives [6].

## 2. Preliminaries and Definitions

The three most common definitions for fractional derivative can be given as the Grünwald-Letnikov definition, the Riemann-Liouville definition, and the Caputo definition.

**Definition 1**

The Riemann-Liouville type fractional integral of order *α* > 0 of a function *f* : (0, *∞*) → *R* is defined by

(1) $$\begin{array}{c} I^{\alpha }f\left(t\right)=\frac{1}{\Gamma \left(\alpha \right)}\overset{t}{\underset{0}{\int }}{\left(t-\tau \right)}^{\alpha -1}f\left(\tau \right)d\tau , \end{array}$$

where Γ(·) is the gamma function.

**Definition 2**

The Grünwald-Letnikov definition is given as

(2) $$\begin{array}{c} {\;}_{a}D_{t}^{\alpha }f\left(t\right)=\underset{}{\underset{h\to 0}{\lim}h^{-\alpha }}\underset{j=0}{\overset{\left[\left(t-a\right)/h\right]}{\sum }}{\left(-1\right)}^{j}\left(\begin{array}{c} \alpha \\ j \end{array}\right)f\left(t-jh\right). \end{array}$$

**Definition 3**

The Riemann-Liouville type fractional derivative of order *α* > 0 of a function *f* : (0, *∞*) → *R* is defined by

(3) $$\begin{array}{c} D^{\alpha }f\left(t\right)=\frac{d^{n}}{dt^{n}}\frac{1}{\Gamma \left(n-\alpha \right)}\overset{t}{\underset{0}{\int }}{\left(t-\tau \right)}^{n-\alpha -1}f\left(\tau \right)d\tau , \end{array}$$

where *n* = [*α*] + 1 and [*α*] is the integer part of *α*.

**Definition 4**

The Caputo type fractional derivative of order *α* > 0 of a function *f* : (0, *∞*) → *R* is defined by

(4) $$\begin{array}{c} D^{\alpha }f\left(t\right)=\frac{1}{\Gamma \left(n-\alpha \right)}\overset{t}{\underset{0}{\int }}{\left(t-\tau \right)}^{n-\alpha -1}f^{n}\left(\tau \right)d\tau , \end{array}$$

where *n* = [*α*] + 1 and [*α*] is the integer part of *α*.

Some properties of the Caputo derivative and the Riemann-Liouville derivative formulas are given below:

(5) $$\begin{array}{c} {\;}_{a}^{C}D_{t}^{\alpha }\left({\;}_{a}^{C}D_{t}^{m}f\left(t\right)\right)={\;}_{a}^{C}D_{t}^{m}\left({\;}_{a}^{C}D_{t}^{\alpha }f(t)\right)={\;}_{a}^{C}D_{t}^{\alpha +m}f\left(t\right),\\ f^{\left(s\right)}\left(0\right)=0,\;s=n,n+1,\ldots ,m\\ m=0,1,2,\ldots ;\;n-1<\alpha <n,\\ {\;}_{a}D_{t}^{m}\left({\;}_{a}D_{t}^{\alpha }f\left(t\right)\right)={\;}_{a}D_{t}^{\alpha }\left({\;}_{a}D_{t}^{m}f(t)\right)={\;}_{a}D_{t}^{\alpha +m}f\left(t\right),\\ f^{\left(s\right)}\left(0\right)=0,\;s=0,1,2,\ldots ,m\\ m=0,1,2,\ldots ;\;n-1<\alpha <n. \end{array}$$

We see that, contrary to the Riemann-Liouville approach, in the case of the Caputo derivative, there are no restrictions on the values *f* ^(*s*)^(0) (*s* = 0,1 …, *n* − 1).

## 3. Equilibrium Points and Their Locally Asymptotic Stability

In this section, we consider a fractional-order nonlinear two-dimensional system as follows:

(6) $$\begin{array}{c} D^{2\alpha }x_{1}\left(t\right)=-\alpha_{1}x_{1}+\beta_{1}x_{2}\left(1-\varepsilon x_{2}^{2}\right)+A_{1},\\ D^{2\alpha }x_{2}\left(t\right)=-\alpha_{2}x_{2}+\beta_{2}x_{1}\left(1-\varepsilon x_{1}^{2}\right)+A_{2},\\ x_{1}\left(0\right)=0,\;\;x_{2}\left(0\right)=0, \end{array}$$

where *D* ^2*α*^ is the fractional derivative of order 1 < 2*α* ≤ 2.  *α*_*i*_ > 0, *α*_*i*_, *β*_*i*_, and *A*_*i*_ (*i* = 1,2) are real constants. These parameters are oblivion, reaction, and attraction constants. In the equations above, we assume that feelings decay exponentially fast in the absence of partners. The parameters specify the romantic style of individuals 1 and 2. In the beginning of relationships, because they have no feelings towards each other, initial conditions are considered zero.

We note that, with zero initial conditions, the following equation is valid:

(7) $$\begin{array}{c} D^{\alpha }\left(D^{\alpha }x\left(t\right)\right)=D^{2\alpha }\left(x\left(t\right)\right). \end{array}$$

In that case, the system can be considered as follows:

(8) $$\begin{array}{c} D^{2\alpha }x_{1}\left(t\right)=D^{\alpha }\left(D^{\alpha }x_{1}\left(t\right)\right),\\ D^{2\alpha }x_{2}\left(t\right)=D^{\alpha }\left(D^{\alpha }x_{2}\left(t\right)\right),\\ x_{1}\left(0\right)=0,\;\;x_{2}\left(0\right)=0. \end{array}$$

Let us make the following changes of variables:

(9) $$\begin{array}{c} x_{1}=y_{1},\;\;D^{\alpha }x_{1}=y_{2},\\ x_{2}=y_{3},\;\;D^{\alpha }x_{2}=y_{4}. \end{array}$$

Then, transformed system is given below:

(10) Dαy1(t)=y2,Dαy2(t)=−α1y1+β1y3(1−εy32)+A1,Dαy3(t)=y4,Dαy4(t)=−α2y3+β2y1(1−εy12)+A2,

with initial conditions

(11) $$\begin{array}{c} y_{1}\left(0\right)=0,\;\;y_{2}\left(0\right)=0,\;\;y_{3}\left(0\right)=0,\;\;y_{4}\left(0\right)=0, \end{array}$$

where 0.5 < *α* ≤ 1, *α*_*i*_ > 0, *α*_*i*_, *β*_*i*_, and *A*_*i*_ (*i* = 1,2) are real constants.

Let *α* ∈ (0.5,1] and consider the system

(12) $$\begin{array}{c} D^{\alpha }y_{1}\left(t\right)=f_{1}\left(y_{1},y_{2},y_{3},y_{4}\right),\\ D^{\alpha }y_{2}\left(t\right)=f_{2}\left(y_{1},y_{2},y_{3},y_{4}\right),\\ D^{\alpha }y_{3}\left(t\right)=f_{3}\left(y_{1},y_{2},y_{3},y_{4}\right),\\ D^{\alpha }y_{4}\left(t\right)=f_{4}\left(y_{1},y_{2},y_{3},y_{4}\right), \end{array}$$

with the initial values

(13) $$\begin{array}{c} y_{1}\left(0\right)=0,\;\;y_{2}\left(0\right)=0,\;\;y_{3}\left(0\right)=0,\;\;y_{4}\left(0\right)=0. \end{array}$$

Here,

(14) f1(y1,y2,y3,y4)=y2,f2(y1,y2,y3,y4)=−α1y1+β1y3(1−εy32)+A1,f3(y1,y2,y3,y4)=y4,f4(y1,y2,y3,y4)=−α2y3+β2y1(1−εy12)+A2.

To evaluate the equilibrium points, let

(15) $$\begin{array}{c} D^{\alpha }y_{i}\left(t\right)=0\Rightarrow f_{i}\left(y_{1}^{*},y_{2}^{*},y_{3}^{*},y_{4}^{*}\right)=0,\;i=1,2,3,4, \end{array}$$

from which we can get the equilibrium points *K*_0_ = (0,0, 0,0) for *A*_1_ = *A*_2_ = 0 and *K*_1_ = (*y*_1_*, *y*_2_*, *y*_3_*, *y*_4_*).

The Jacobian matrix *J*(*K*_1_) for the system given in (14) is

(16) $$\begin{array}{c} J\left(K_{1}\right)=\left[\begin{array}{cccc} 0 & 1 & 0 & 0\\ -\alpha_{1} & 0 & a & 0\\ 0 & 0 & 0 & 1\\ b & 0 & -\alpha_{2} & 0 \end{array}\right], \end{array}$$

where

(17) $$\begin{array}{c} a=\beta_{1}\left(1-3\varepsilon {\left(y_{3}^{*}\right)}^{2}\right),\;\;b=\beta_{2}\left(1-3\varepsilon y_{1}^{*2}\right). \end{array}$$

To discuss the local stability of the equilibrium *K*_1_ = (*y*_1_*, *y*_2_*, *y*_3_*, *y*_4_*) of the system given by (14), we consider the linearized system at *K*_1_. The characteristic equation of the linearized system is of the form

(18) $$\begin{array}{c} P\left(\lambda \right)=\lambda^{4}+\left(\alpha_{2}+\alpha_{1}\right)\lambda^{2}+\left(\alpha_{1}\alpha_{2}-ab\right)=0. \end{array}$$

If *λ* ^2^ is taken as *k*, we have the following reduced equation:

(19) $$\begin{array}{c} P\left(\lambda \right)=k^{2}+a_{1}k+a_{2}=0, \end{array}$$

where

(20) $$\begin{array}{c} a_{1}=\left(\alpha_{2}+\alpha_{1}\right),\\ a_{2}=\left(\alpha_{1}\alpha_{2}-ab\right). \end{array}$$

According to the fractional Routh-Hurwitz criteria, we have the following theorem.

**Theorem 5**

If *a*_1_ > 0 and *a*_2_ > 0, then the equilibrium point *K*_1_ = (*y*_1_*, *y*_2_*, *y*_3_*, *y*_4_*) is locally asymptotically stable for all *α* ∈ (0,1).

**Proof**

*K*_1_ = (*y*_1_*, *y*_2_*, *y*_3_*, *y*_4_*) equilibrium of the system given by (12) is asymptotically stable if all of the eigenvalues, *k*_*i*_, *i* = 1,2, of *J*(*K*_1_), satisfy the following condition (negative real part) [7, 8]:

(21) $$\begin{array}{c} \left|\arg\lambda_{i}\right|>\frac{\alpha \pi }{2}. \end{array}$$

For *n* = 2, the Routh-Hurwitz criteria are just *a*_1_ > 0 and *a*_2_ > 0. The characteristic polynomial *P*(*λ*) = *k* ^2^ + *a*_1_ *k* + *a*_2_ = 0 satisfies eigenvalues as below:

(22) $$\begin{array}{c} k_{1,2}=\frac{-a_{1}\pm \sqrt{a_{1}^{2}-4a_{2}}}{2}. \end{array}$$

Now, suppose that *a*_1_ and *a*_2_ are positive. It is easy to see that if the roots are real, they are both negative, and if they are complex conjugates, they have a negative real part.

Next, to prove the converse, suppose that the roots are either negative or have a negative real part. Then, it follows that *a*_1_ > 0. If the roots are complex conjugates, 0 < *a*_1_^2^ < 4*a*_2_, which implies that *a*_2_ is also positive. If the roots are real, then since both of the roots are negative, it follows that *a*_2_ > 0.

**Theorem 6**

Let *a*_2_ = (*α*_1_ *α*_2_ − *ab*) be as given in (20). If *a*_2_ < 0, then the positive equilibrium point *K*_1_ = (*y*_1_*, *y*_2_*, *y*_3_*, *y*_4_*) of the system given in (12) is unstable.

**Proof**

If *a*_2_ < 0, from Descartes' rule of signs, it is clear that the characteristic equation *P*(*λ*) has at least one positive real root. So, the equilibrium point *K*_1_ = (*y*_1_*, *y*_2_*, *y*_3_*, *y*_4_*) of the system given in (12) is unstable.

## 4. Analysis of a Model with Upper Bounds

In this section, we consider fractional-order system with the order *α* between 0 < *α* < 1:

(23) $$\begin{array}{c} D^{\alpha }x_{1}\left(t\right)=-\alpha_{1}x_{1}+\beta_{1}x_{2}\left(1-\varepsilon x_{2}^{2}\right)+A_{1},\\ D^{\alpha }x_{2}\left(t\right)=-\alpha_{2}x_{2}+\beta_{2}x_{1}\left(1-\varepsilon x_{1}^{2}\right)+A_{2},\\ x_{1}\left(0\right)=0,\;\;x_{2}\left(0\right)=0. \end{array}$$

A detailed analysis of this model is given in [5]. With the help of the following lemmas, upper bounds are discussed for the system.

Before giving our results, we give some useful lemmas [9, 10].

**Lemma 7**

Let *α*, *β*, *γ*, and *p* be positive constants. Then,

(24) ∫0t(t−s)p(β−1)sp(γ−1)ds =tθB[p(γ−1)+1,p(β−1)+1], t∈R+,

where *B*[*ξ*, *η*] = ∫_0_^1^ *s* ^*ξ*−1^(1 − *s*)^*η*−1^ *ds*(*ℜξ* > 0,*ℜη* > 0) ve *θ* = *p*(*β* + *γ* − 2) + 1.

**Lemma 8**

Let *u*, *v*, and *f*_*i*_ ∈ *C*(*I*, *R*_+_), *i* = 1,2, with *f*_*i*_ be nondecreasing; let *φ*_*ij*_ ∈ *C*(*I* × *I*, *R*_+_) be nondecreasing in a variable *t* for every *s* fixed (*i* = 1,2). If

(25) u(t)≤f1(t)+∫0t[φ11(t,s)u(s)+φ12(t,s)v(s)]ds,v(t)≤f2(t)+∫0t[φ21(t,s)u(s)+φ22(t,s)v(s)]ds,                   t∈I,

then, for *t* ∈ *I*, one has

(26) u(t)≤[f1(t)+f2(t)∫0tφ12(t,s)Φ2(s)ds]×exp⁡{∫0tφ11(t,s)ds   +∫0tφ12(t,s)Φ2(s)      ×(∫0sφ21(s,τ)Φ1(τ)dτ)ds},v(t)≤[f2(t)+f1(t)∫0tφ21(t,s)Φ1(s)ds]×exp⁡{∫0tφ22(t,s)ds    +∫0tφ21(t,s)Φ1(s)       ×(∫0sφ12(s,τ)Φ2(τ)dτ)ds},

where Φ_*i*_(*t*): = exp⁡∫_0_^*t*^ *φ*_*ii*_(*t*, *s*)*ds*, *i* = 1,2.

Let *α* ∈ (0,1] and consider the system

(27) $$\begin{array}{c} D^{\alpha }x_{1}\left(t\right)=f_{1}\left(t,x_{1},x_{2}\right),\\ D^{\alpha }x_{2}\left(t\right)=f_{2}\left(t,x_{1},x_{2}\right), \end{array}$$

with the initial conditions *x*_1_(0) = 0 and *x*_2_(0) = 0. Here, *f*_1_(*t*, *x*_1_, *x*_2_) = −*α*_1_ *x*_1_ + *β*_1_ *x*_2_(1 − *εx*_2_^2^) + *A*_1_ and *f*_2_(*t*, *x*_1_, *x*_2_) = −*α*_2_ *x*_2_ + *β*_2_ *x*_1_(1 − *εx*_1_^2^) + *A*_2_. Now, upper bounds for a fractional-order nonlinear system are discussed with the following theorem.

**Theorem 9**

Let *f*_1_ and *f*_2_ ∈ *C*(*I* × *R* ^2^, *R*) and satisfy the following inequality:

(28) $$\begin{array}{c} \left|f_{1}\left(t,x_{1},x_{2}\right)\right|\leq \alpha_{1}\left(t\right)\left|x_{1}\right|+\beta_{1}\left(t\right)\left|x_{2}\right|,\\ \left|f_{2}\left(t,x_{1},x_{2}\right)\right|\leq \beta_{2}\left(t\right)\left|x_{1}\right|+\alpha_{2}\left(t\right)\left|x_{2}\right|, \end{array}$$

where *α*_*i*_ and *β*_*i*_ ∈ *C*(*I*, *R*_+_) (*i*, *j* = 1,2) and *x*_1_,*x*_2_ ∈ *R*. Then, one has the following upper bounds for system of fractional order:

(29) |x1(t)| ≤tα−1  ×exp⁡{1qk∗(t)     ×[∫0tα1q(s)ds       +∫0tβ1q(s)Ψ2(s)         ×(k∗(s)∫0sβ2q(τ)Ψ1(τ)dτ)ds]},|x2(t)| ≤tα−1  ×exp⁡{1qk∗(t)     ×[∫0tα2q(s)ds      +∫0tβ2q(s)Ψ1(s)        ×(k∗(s)∫0sβ1q(τ)Ψ2(τ)dτ)ds]},

for *t* > 0, where

(30) $$\begin{array}{c} p=\frac{1+4\alpha }{1+3\alpha },\;\;q=\frac{1+4\alpha }{\alpha },\\ k^{*}\left(t\right)=\frac{t^{q\alpha -1}B^{q/p}\left[p\left(\alpha -1\right)+1,p\left(\alpha -1\right)+1\right]}{\Gamma^{q}\left(\alpha \right)},\\ \Psi_{1}\left(t\right)=\exp(k^{*}\left(t\right)\overset{t}{\underset{0}{\int }}\alpha_{1}^{q}\left(s\right)ds),\\ \Psi_{2}\left(t\right)=\exp(k^{*}\left(t\right)\overset{t}{\underset{0}{\int }}\alpha_{2}^{q}\left(s\right)ds). \end{array}$$

**Proof**

Since *f*_1_ and *f*_2_ are assumed to be continuous functions, every solution of the initial value problem (IVP) given by (23) is also a solution of the following integral system for 0 < *α* < 1:

(31) $$\begin{array}{c} x_{1}\left(t\right)=\frac{1}{\Gamma \left(\alpha \right)}\overset{t}{\underset{0}{\int }}{\left(t-\tau \right)}^{\alpha -1}f_{1}\left(\tau ,x_{1}\left(\tau \right),x_{2}\left(\tau \right)\right)d\tau ,\\ x_{2}\left(t\right)=\frac{1}{\Gamma \left(\alpha \right)}\overset{t}{\underset{0}{\int }}{\left(t-\tau \right)}^{\alpha -1}f_{2}\left(\tau ,x_{1}\left(\tau \right),x_{2}\left(\tau \right)\right)d\tau . \end{array}$$

Moreover, every solution of integral system is a solution of the IVP [11]. Now, we derive from (28) and (33) the following:

(32) $$\begin{array}{c} \beta \left(t\right)\leq \frac{t^{1-\alpha }}{\Gamma \left(\alpha \right)}\overset{t}{\underset{0}{\int }}{\left(t-\tau \right)}^{\alpha -1}\tau^{\alpha -1}\left[\alpha_{1}\beta \left(\tau \right)+\beta_{1}\gamma \left(\tau \right)\right]d\tau ,\\ \gamma \left(t\right)\leq \frac{t^{1-\alpha }}{\Gamma \left(\alpha \right)}\overset{t}{\underset{0}{\int }}{\left(t-\tau \right)}^{\alpha -1}\tau^{\alpha -1}\left[\beta_{2}\beta \left(\tau \right)+\alpha_{2}\gamma \left(\tau \right)\right]d\tau , \end{array}$$

where

(33) $$\begin{array}{c} \beta \left(t\right)=\left|x_{1}\left(t\right)\right|t^{1-\alpha },\;\;\gamma \left(t\right)=\left|x_{2}\left(t\right)\right|t^{1-\alpha }. \end{array}$$

Using Hölder's inequality for (1/*p*) + (1/*q*) = 1 with *p* = (1 + 4*α*)/(1 + 3*α*) in (32), we get the inequality below:

(34) $$\begin{array}{c} \beta \left(t\right)\leq \frac{t^{1-\alpha }}{\Gamma \left(\alpha \right)}k\left(t\right){\left(\overset{t}{\underset{0}{\int }}[\alpha_{1}^{q}\beta^{q}(\tau )+\beta_{1}^{q}\gamma^{q}(\tau )]d\tau \right)}^{1/q},\\ \gamma \left(t\right)\leq \frac{t^{1-\alpha }}{\Gamma \left(\alpha \right)}k\left(t\right){\left(\overset{t}{\underset{0}{\int }}[\beta_{2}^{q}\beta^{q}(\tau )+\alpha_{2}^{q}\gamma^{q}(\tau )]d\tau \right)}^{1/q}, \end{array}$$

where *k*(*t*) = (∫_0_^*t*^(*t*−*τ*)^*p*(*α*−1)^ *τ* ^*p*(*α*−1)^ *dτ*)^1/*p*^. Then, we have

(35) $$\begin{array}{c} \beta^{q}\left(t\right)\leq \frac{t^{q(1-\alpha )}}{\Gamma^{q}\left(\alpha \right)}k^{q}\left(t\right)\overset{t}{\underset{0}{\int }}\left[\alpha_{1}^{q}\beta^{q}\left(\tau \right)+\beta_{1}^{q}\gamma^{q}\left(\tau \right)\right]d\tau ,\\ \gamma^{q}\left(t\right)\leq \frac{t^{q(1-\alpha )}}{\Gamma^{q}\left(\alpha \right)}k^{q}\left(t\right)\overset{t}{\underset{0}{\int }}\left[\beta_{2}^{q}\beta^{q}\left(\tau \right)+\alpha_{2}^{q}\gamma^{q}\left(\tau \right)\right]d\tau . \end{array}$$

By Lemma 7, the following inequality is obtained for *k*(*t*):

(36) $$\begin{array}{c} k\left(t\right)=t^{\left(2\alpha -2)+(1/p\right)}B^{1/p}\left[p\left(\alpha -1\right)+1,p\left(\alpha -1\right)+1\right]. \end{array}$$

By using the last relation in (35), we obtain

(37) $$\begin{array}{c} \beta^{q}\left(t\right)\leq k^{*}\left(t\right)\overset{t}{\underset{0}{\int }}\left[\alpha_{1}^{q}\beta^{q}\left(\tau \right)+\beta_{1}^{q}\gamma^{q}\left(\tau \right)\right]d\tau ,\\ \gamma^{q}\left(t\right)\leq k^{*}\left(t\right)\overset{t}{\underset{0}{\int }}\left[\beta_{2}^{q}\beta^{q}\left(\tau \right)+\alpha_{2}^{q}\gamma^{q}\left(\tau \right)\right]d\tau , \end{array}$$

where

(38) $$\begin{array}{c} k^{*}\left(t\right)=\frac{t^{q\alpha -1}B^{q/p}\left[p\left(\alpha -1\right)+1,p\left(\alpha -1\right)+1\right]}{\Gamma^{q}\left(\alpha \right)}. \end{array}$$

Since

(39) $$\begin{array}{c} p\left(\alpha -1\right)+1=\frac{1+4\alpha }{1+3\alpha }\left(\alpha -1\right)+1=\frac{4\alpha^{2}}{1+3\alpha }>0,\\ q\alpha -1=\left(\frac{1+4\alpha }{\alpha }\right)\alpha -1=4\alpha >0, \end{array}$$

we have the following inequality:

(40) $$\begin{array}{c} 0<B\left[p\left(\alpha -1\right)+1,p\left(\alpha -1\right)+1\right]<+\infty \end{array}$$

and the function k*(*t*) is nondecreasing on *I*. Now, with an application of Lemma 8 to (37) combining with (33), upper bounds for a fractional-order nonlinear system are obtained.

## 5. Numerical Simulation

In nonlinear dynamic systems, predictability can be possible with stability. Also relationship development would be predictable given the right parameters. In this model, parameters provide the condition for the locally asymptotic stability of equilibrium points by using stability analysis on fractional-order transformed system.

In this paper, we focus on couple dynamics depending on the parameters as below. Many scenarios are possible. But in this model, secure or cautious lover (individual 1 retreats from his own feelings but is encouraged by that of individual 2 (*α*_1_ < 0 and *β*_1_ > 0)) and hermit (individual 1 retreats from his own feelings and that of individual 2 (*α*_2_ < 0 and *β*_2_ < 0)) are considered.

Let

(41) $$\begin{array}{c} \alpha_{1}=0.005,\;\alpha_{2}=0.006,\;\beta_{1}=0.0004,\\ \beta_{2}=-0.0001,\;\varepsilon =0.001,\;A_{1}=0.02,\\ A_{2}=0.03,\;2\alpha =1.6. \end{array}$$

Now, we consider the system

(42) $$\begin{array}{c} D^{2\alpha }x_{1}=-0.005x_{1}+0.0004x_{2}\left(1-0.001x_{2}^{2}\right)+0.02,\\ D^{2\alpha }x_{2}=-0.0001x_{1}\left(1-0.001x_{1}^{2}\right)-0.006x_{2}+0.03. \end{array}$$

Let the initial conditions be

(43) $$\begin{array}{c} x_{1}\left(0\right)=0,\;\;x_{2}\left(0\right)=0. \end{array}$$

After the system is transformed, the following system is obtained with the order of *α* = 0.8:

(44) f1(y1,y2,y3,y4)=y2,f2(y1,y2,y3,y4)=−0.005y1+0.0004y3(1−0.001y32)+0.02,f3(y1,y2,y3,y4)=y4,f4(y1,y2,y3,y4)=−0.006y3−0.0001y1(1−0.001y12)+0.03.

Let the initial conditions be

(45) $$\begin{array}{c} y_{1}\left(0\right)=0,\;\;y_{2}\left(0\right)=0,\;\;y_{3}\left(0\right)=0,\;\;y_{4}\left(0\right)=0. \end{array}$$

Positive equilibrium point for the problem (44) and (45) is calculated as

(46) $$\begin{array}{c} y_{1}^{*}=4.38469,\;y_{2}^{*}=0,\;y_{3}^{*}=4.92833,\;y_{4}^{*}=0. \end{array}$$

**Figure 1:**
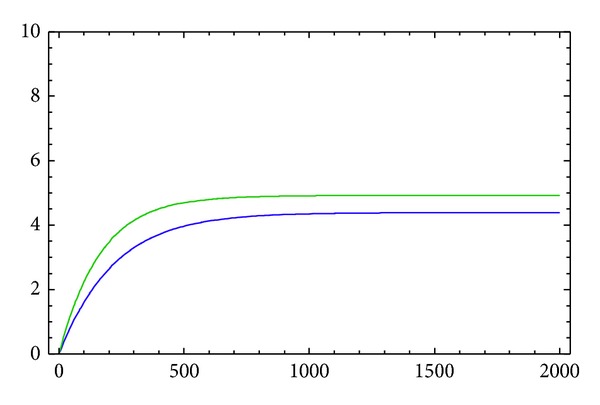
The graphs of *y*_3_(*t*) (above) and *y*_1_(*t*) (below) with the order 2*α*.

**Figure 2:**
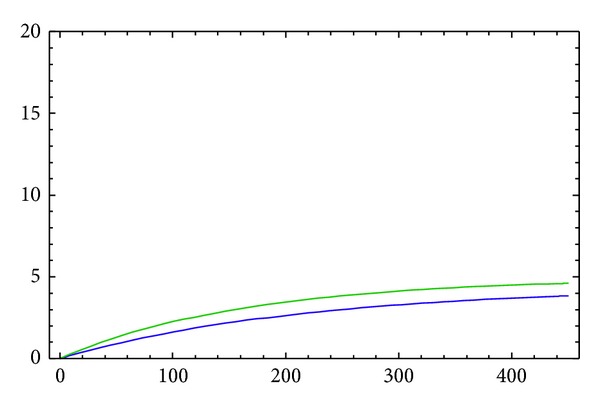
Approximation to the equilibrium point with the order *α*.

The approximate solutions *y*_1_(*t*) and *y*_3_(*t*) (resp., govern the feelings (*x*_1_) of *A* to *B* and the feelings (*x*_2_) of *B* to *A*) are displayed in Figure 1 for 2*α* = 1.6 with acceleration in feelings.  Figure 2 shows the asymptotic approximation of (*y*_1_(*t*), *y*_2_(*t*), *y*_3_(*t*), *y*_4_(*t*)) to the equilibrium point (4.38469,0, 4.92833,0) for *α* = 0.8. For the numerical solution of the system, we use the predictor corrector method [12].

We have demonstrated via numerical simulations that the fractional-order nonlinear couple model (42) and (43) can exhibit asymptotic behavior in the presence of nonlinearity for an appropriate set of model parameters. We have observed that the model approaches the equilibrium points asymptotically.

## 6. Conclusions

In this paper, stability analysis of the fractional-order nonlinear dynamical model of couple is studied by using the fractional Routh-Hurwitz criteria. By using stability analysis on fractional-order system, sufficient condition on the parameters for the locally asymptotic stability of equilibrium points is obtained. A fractional-order nonlinear dynamical model of couple with the order 2*α* has been formulated and analyzed. In the discussed model, acceleration is observed in the solution. Also upper bounds for a system with the order *α* have been obtained.

Finally, we have demonstrated via numerical simulations that a fractional-order nonlinear model of couple can exhibit asymptotic behavior in the presence of an appropriate set of model parameters.

## References

[1] Machado JT, Kiryakova V, Mainardi F (2011). Recent history of fractional calculus. *Communications in Nonlinear Science and Numerical Simulation*.

[2] Barley K, Cherif A (2011). Stochastic nonlinear dynamics of interpersonal and romantic relationships. *Applied Mathematics and Computation*.

[3] Gottman JM, Murray JD, Swanson CC, Tyson R, Swanson KR (2002). *The Mathematics of Marriage*.

[4] Martin TC, Bumpass LL (1989). Recent trends in marital disruption. *Demography*.

[5] Ozalp N, Koca I (2012). A fractional order nonlinear dynamical model of interpersonal relationships. *Advances in Difference Equations*.

[6] Podlubny I (1999). *Fractional Differential Equations*.

[7] Ahmed E, El-Sayed AMA, El-Saka HAA (2007). Equilibrium points, stability and numerical solutions of fractional-order predator-prey and rabies models. *Journal of Mathematical Analysis and Applications*.

[8] Matignon D (1996). Stability results for fractional differential equations with applications to control processing. *Computational Engineering in Systems and Application Multiconference*.

[9] Ma Q-H, Yang EH (2002). Some new Gronwall-Bellman-Bihari type integral inequalities with delay. *Periodica Mathematica Hungarica*.

[10] Prudnikov AP, Brychkov YA, Marichev OL (1981). *Integrals and Series of Elementary Functions*.

[11] Lakshmikantham V, Vatsala AS (2007). Theory of fractional differential inequalities and applications. *Communications in Applied Analysis*.

[12] Diethelm K, Ford NJ, Freed AD (2002). A predictor-corrector approach for the numerical solution of fractional differential equations. *Nonlinear Dynamics*.

